# Ordered Monolayer Gold Nano-urchin Structures and Their Size Induced Control for High Gas Sensing Performance

**DOI:** 10.1038/srep24625

**Published:** 2016-04-19

**Authors:** Ylias M. Sabri, Ahmad Esmaielzadeh Kandjani, Samuel J. Ippolito, Suresh K. Bhargava

**Affiliations:** 1 Centre for Advanced Materials and Industrial Chemistry (CAMIC), School of Applied Sciences, RMIT University, GPO Box 2476V, Melbourne, VIC 3001 (Australia)

## Abstract

The synthesis of ordered monolayers of gold nano-urchin (Au-NU) nanostructures with controlled size, directly on thin films using a simple electrochemical method is reported in this study. In order to demonstrate one of the vast potential applications, the developed Au-NUs were formed on the electrodes of transducers (QCM) to selectively detect low concentrations of elemental mercury (Hg^0^) vapor. It was found that the sensitivity and selectivity of the sensor device is enhanced by increasing the size of the nanospikes on the Au-NUs. The Au-NU-12 min QCM (Au-NUs with nanospikes grown on it for a period of 12 min) had the best performance in terms of transducer based Hg^0^ vapor detection. The sensor had 98% accuracy, 92% recovery, 96% precision (repeatability) and significantly, showed the highest sensitivity reported to date, resulting in a limit of detection (LoD) of only 32 μg/m3 at 75 °C. When compared to the control counterpart, the accuracy and sensitivity of the Au-NU-12 min was enhanced by ~2 and ~5 times, respectively. The results demonstrate the excellent activity of the developed materials which can be applied to a range of applications due to their long range order, tunable size and ability to form directly on thin-films.

Ordered nanostructures have been widely employed to develop many different types of devices for various applications including biosensors^1^,^2^, optical devices^3^,^4^, data storage^5^,^6^,^7^ and lithography just to name a few^8^,^9^,^10^,^11^,^12^. Recently, there have been a large number of reports on many different ordered nanostructures with various shapes, sizes and properties, only possible as a result of the progress and implementation of great many different techniques^13^. However, the forthcoming challenge is to hybridize nano-materials with ordered nanostructures and tailorable functions to develop high performance devices for numerous applications. In order to produce ordered nanostructures on a particular device, patterning techniques that allow for high degree of precision regarding the alignments, position, shape, size and spacing, need to be employed. Moreover, the nano-patterning techniques need to enable the patterning of large areas, have high throughput and be cost effective at the same time. There have been many efforts to obtain ordered nanostructures using electron-beam lithography (EBL)^14^,^15^, focused ion beam (FIB)^16^, nanoimprint lithography (NIL)^17^, mask lithography (*via* porous alumina)^18^ and self-assembly^19^. Although top-down approaches such as EBL and FIB milling^16^ allow for the creation of regular arrays of nanostructured patterns, they require costly equipment, have slow turnaround time, and are difficult to use for patterning large substrates. On the other hand, self-assembling approaches using the bottom-up techniques have also aroused great attention due to the ease of producing periodic nanostructures and the richness of the results that can be offered^7^,^20^. An example of such technique is the self-assembly of polystyrene (PS) nanospheres being used as a template to produce a variety of devices due to the possibility of achieving large-scale coverage and uniformity, excellent reproducibility, fast processing and the low fabrication cost involved^21^,^22^,^23^. Furthermore, self-assembling can be applied to numerous organic, inorganic and metallic materials thereby creating a range of close packed monodispersed nanostructures that can be used as nanolasers, optical filters, waveguides and chemical sensors *etc*^3^,^13^,^24^,^25^. The most important aspect of using close packed monodispersed nanostructures in chemical sensing applications, for example, lies in their periodic structures which are the key to harnessing better repeatability, sensitivity and accuracy^13^,^20^,^24^,^26^.

One of the most important chemical sensing applications that is currently sought-after by the scientific community around the world are mercury sensors^27^,^28^,^29^,^30^,^31^,^32^,^33^. Among the different species of mercury, elemental mercury (Hg^0^) vapor represents 66–94% of emitted from anthropogenic sources^34^,^35^. The urgency of detecting Hg^0^ vapor from anthropogenic emission sources arises from the fact that the toxin can settle in oceans, convert to its deadliest form (*i.e.* organic forms) before it is bioaccumulated in fish thereby entering the food chain^36^. Therefore, the detection of Hg^0^ vapor at the emission source is the first step in reducing emissions through evaluating the efficiency of mercury removal technologies implemented in industrial processes. Mercury measurements are typically performed by commercially available atomic absorption (AA) or atomic fluorescence (AF) spectrometry based systems. Although these techniques are highly sensitive with low limit of detection (LoD), there are several major issues associated with these systems. Some of the issues with these techniques are arisen as they are based on Hg^0^ undergoing absorption/emission at 253.7 nm wavelength which can cause the occurrence of undesired photochemical reactions in the industrial effluent streams which contain other interfering gas species that are excited by the same UV wavelength. Furthermore, these systems are costly, bulky, require specialized sample collection and gas conditioning as well as being associated with many challenges before they can be employed as online sensors^28^,^37^,^38^,^39^,^40^. In addition to the aforementioned limitation of the analytical instruments, the sampling requirements associated with the Ontario Hydro (OH) or Appendix K methods prior to detection are also expensive, require skilled operators and can have up to 2-week turnaround time thus providing no real-time data^41^. These shortcomings as well as the introduction of more stringent emission regulations introduced by environmental and government bodies (*i.e.* US-EPA, UNEP) have forced many industries (*i.e.* Alumina, Coal, cement, petroleum) to actively pursue alternative methods for measuring Hg^0^ within their gas effluents^41^,^42^,^43^. The attention has now shifted toward developing non-spectroscopic based Hg^0^ vapor sensors which are based on microsensors as they are small, cheap, and robust and they can generally operate continuously with minimal maintenance requirements. Among the non-spectroscopic based Hg^0^ vapor sensors reported to date, our group have shown that quartz crystal microbalance (QCM) transducers are excellent candidates for detecting mercury vapor in industrial effluents. Interestingly, recent studies have found that among other applications^44^,^45^, gold nanostructures can be modified to exhibit extraordinarily high activity when used in Hg^0^ vapor sensor devices^42^. These findings are driving the recent interest in Au chemistry and Au-based nanomaterials to develop transducer based devices that have LoDs comparative to the commercially available spectroscopic systems.

Lately, gold nano-urchins have been reported to have extraordinary properties such as distinct and size-dependent tunable surface plasmon resonance (SPR) bands as well as morphological surface defects generating ‘hot-spots’ essential for surface enhanced Raman spectroscopy (SERS) based sensing applications^46^,^47^,^48^,^49^. However, these structures have so far only been successfully synthesized in the form of colloids thereby limiting their use for thin-film based applications including optics, catalysis, batteries, electrocatalysis, photo-catalysis and chemical/bio-chemical sensing. Although nano-urchin films of semiconductor (*i.e.* ZnO^50^, Au-ZnO^51^ and TiO_2_^52^) nanostructures have been successfully synthesized, a simple method for producing gold nano-urchin monolayer is yet to be demonstrated. Common methods for producing gold urchins are based on colloidal formation of individual Au urchin structures in a liquid solution. Such strategy is good for colloidal studies. However in order to use these materials on substrates with repeatable packing arrangement, the formation of close-packed array prior to nanourchin formation is important. This is because the urchin spikes, if made in colloidal solutions, would otherwise deplete the close packing of the urchins due to 3D morphological hindrance. The novel approach which is introduced in this work overcomes this issue as the nanourchins are formed following the formation of packed monolayer of monodispersed PS templates which ensures achieving close packed and ordered nanourchin structure over large surfaces.

Here, we report gold nano-urchins monolayer (Au-NU) synthesized directly on thin-film transducer devices for non-spectroscopic based (*i.e.* transducer based) Hg^0^ vapor sensing. The importance of the technique introduced in this study comes from the ease in which close packing formation of gold nano-urchin structures are achieved on the substrate surface. When the Au-NUs were formed on the quartz crystal microbalance (QCM) transducers and employed as Hg^0^ vapor sensors, the modified sensors showed >15 times response magnitude relative to a control gold consisting of 100 nm Au film evaporated on the electrode of a QCM transducer (referred to as Au-control). To the best of the authors’ knowledge, this is the most sensitive QCM based mercury vapor sensor reported to date and is a step forward in developing a non-spectroscopic, transducer based mercury vapor sensor for industrial and environmental applications.

## Results and Discussion

**Figure 1:**
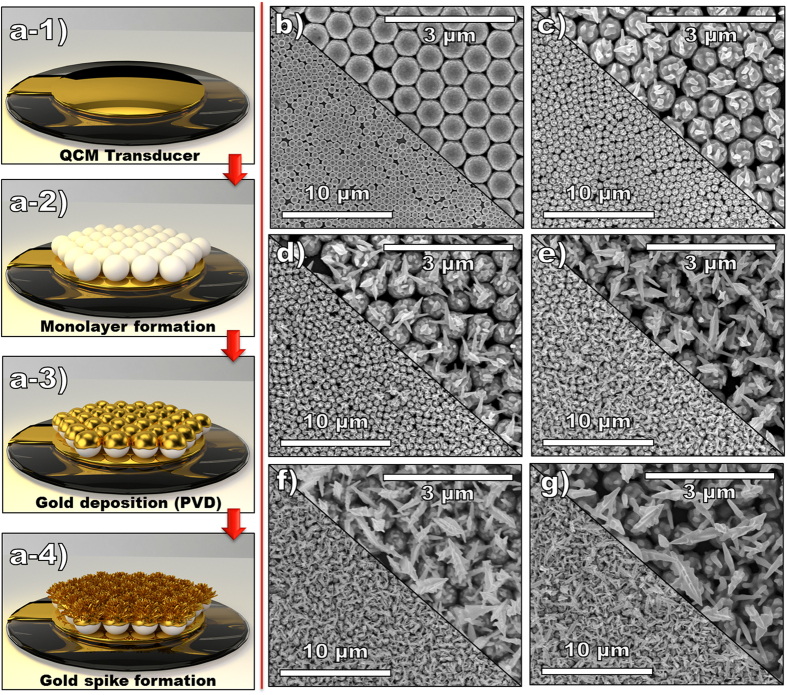
(**a**) Schematic representing the QCM transducer modification process a-1) Depositon of Ti electrodes on quartz substrates through e-beam evaporation in order to form Ti QCMs followed by a-2) the transfer of self-assembled PSNS on the Ti electrodes of the QCMs followed by a-3) deposition of gold through e-beam evaporation to develop Au-MNM and finally a-4) electrochemical deposition of nanospikes to form gold nano-urchins (Au-NUs); and SEM images of (**b**) close-packed Au-MNM, (**c**) Au-NU-6 min, (**d**) Au-NU-8 min, (**e**) Au-NU-10 min, (**f**) Au-NU-12 min and (**g**) Au-NU-15 min, all deposited directly on the Ti electrodes of the QCM transducers.

**Figure 2:**
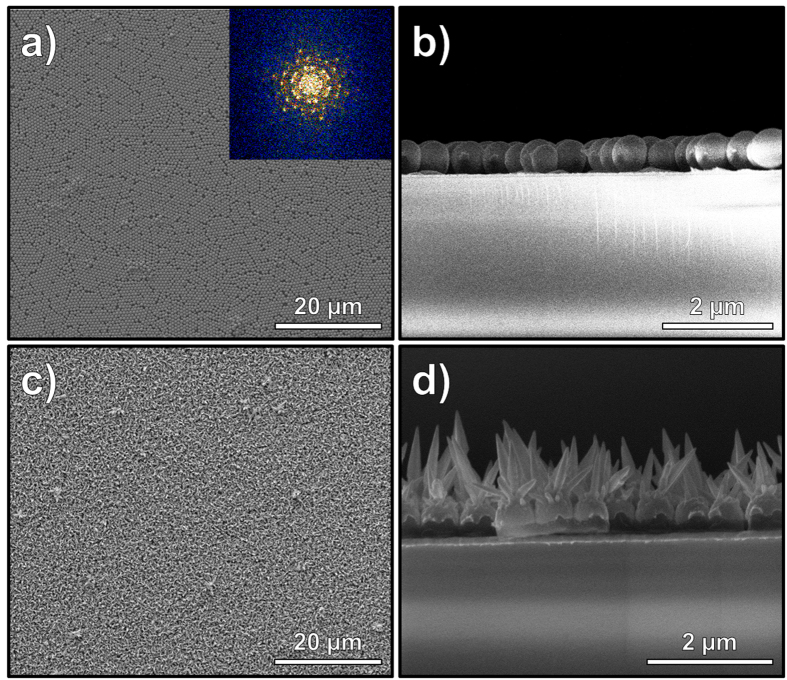
(**a**) Low magnification SEM image for Au-MNM, the inset shows FFT graph of the SEM image; (**b**) Side view of packed monolayer of Au-MNM; (**c**) Low magnification image of Au-NU-12 min sample showing large surface coverage; (**d**) Side view of packed monolayer of Au-NU-12 min showing the formation of nano-urchins.

**Figure 3:**
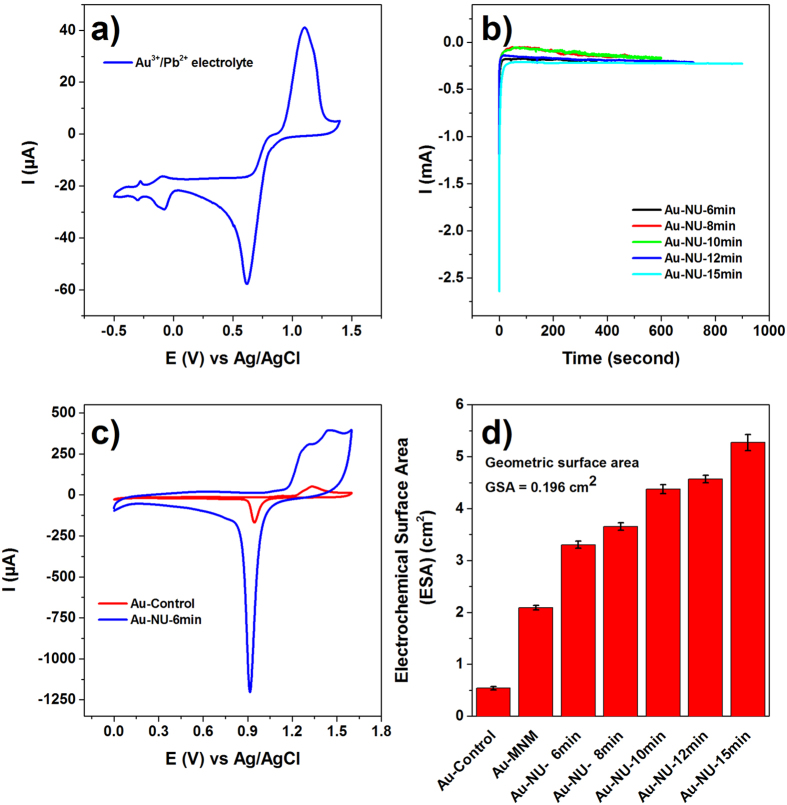
Electrochemical characterization data showing (**a**) cyclic voltammograms (CVs) obtained at an Au electrode for the reduction of gold from an electrolyte containing 2.718 g/L HAuCl_4_•3H_2_O and 0.177 g/L Pb(CH_3_COO)_2_•3H_2_O recorded at 50 mV s^−1^; (**b**) the current stability during the formation of Au-NU with different nanospike sizes; (**c**) Linear sweep voltammograms (LSVs) for the Au-control and Au-NU-6 min samples obtained in 1 M H_2_SO_4_ at 100 mV s^−1^ (**d**) the electrochemical surface area (ESA) calculate from the reduction of one oxide monolayer formed on the Au surfaces during the reduction phase of the cyclic voltammogram recorded in 1 M H_2_SO_4_. The geometric surface area of each substrate was 0.196 cm^2^.

**Figure 4:**
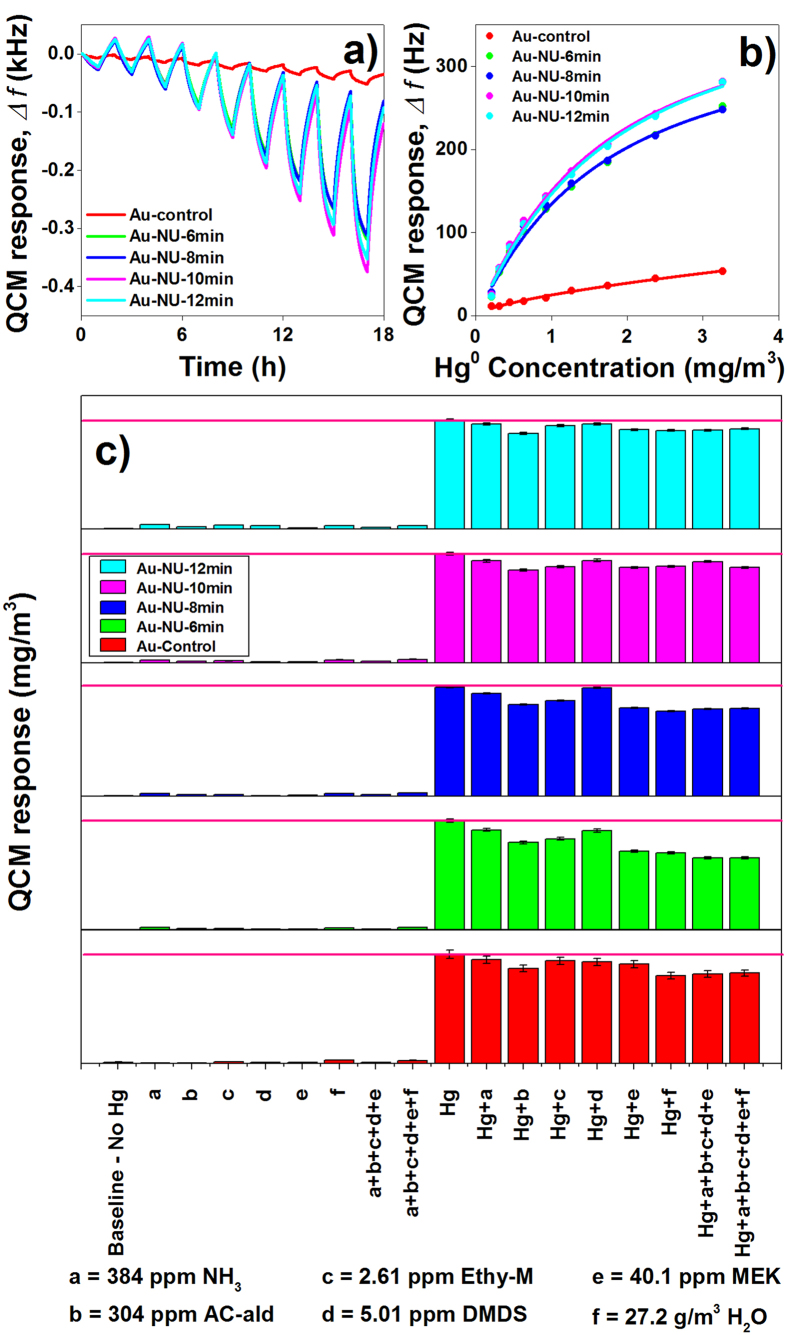
Modified (Au-NUs) and Au-control based QCMs’ (**a**) dynamic response, (**b**) response magnitudes (The solid fitted lines represent the best fits for the LRC equation) and (**c)** selectivity performance toward Hg^0^ vapor at 75 °C. The dynamic response was obtained when the QCMs were exposed toward Hg^0^ vapor concentrations of 0.21, 0.31, 0.45, 0.64, 0.93, 1.27, 1.74, 2.38, and 3.26 ± 0.05 mg/m^3^. The target concentration of 3.26 mg/m^3^ is represented by the solid magenta lines in each of the panels.

Figure 1a shows the summary of the fabrication process of Au-NU directly on QCM transducers. Briefly, a 300 nm Ti film was e-beam evaporated on each of the two sides of the optically polished quartz substrates to form the two electrodes required for oscillation of the crystal (Fig. 1a-1). A monolayer of monodispersed polystyrene nanospheres (PSNS) was deposited directly on the QCM substrates (Fig. 1a-2) and a 100 nm Au film deposition using e-beam evaporation was then followed (Fig. 1a-3) thereby forming gold evaporated monodispersed nanosphere monolayer (Au-MNM). One QCM having no PSNS was also fabricated as the control device and is referred to as Au-control. The Au-NUs were then formed following a one-step electrodeposition of gold nanospikes on the Au-MNM (Fig. 1a-4) for various periods of time. Figure 1b shows the SEM image of the Au-MNM deposited directly on the QCM transducers. The PSNS are observed to be monodispersed, have size of ~500 nm and have formed long-range ordered hexagonally close-packed monolayer on the QCM transducer’s Ti electrode. The SEM images of Au-NU having different sizes are shown in Fig. 1c–g. The different sizes of spikes on the nano-urchins were formed by electrochemical deposition of Au-nanospikes for a period of 6 (**c**), 8 (**d**), 10 (**e**), 12 (**f**) and 15 (**g**) minutes. It can be observed that as the electrochemical deposition time increased, the size of the nanospikes forming nano-urchins also increased reaching sizes of 300 nm and 1.5 μm for deposition times of 6 and 15 minutes, respectively. It is also observed that by increasing the deposition time, the electrodeposited Au nanostructures tend to grow to more defined spikes with relatively sharper tips and with saw-teeth shaped edges and increasing base size. These observations are better demonstrated in the higher SEM magnification of each sample that is shown in the Supplementary information (Fig. S1). The monolayer order packing quality and the growth structure of the nanospikes on Au-MNM monolayers, low magnification and side view SEM images are shown in Fig. 2. In Fig. 2a, the packed monolayer formation of PS monodispersed nanospheres is demonstrated where only minor packing faults observed due to small variations in size as well as some drying effects that occurred during monolayer formation process. The successful formation of a single layer of close-packed PSNS can be clearly seen from Fig. 2b where a side view SEM image of Au-MNM is presented. Although some packing faults have induced discrete close packed islands, using a fast Fourier transformation (FFT) showed that distinct and uniform hexagonal spots have been formed on the substrate (see inset in Fig. 2a)^53^. The low magnification SEM image for Au-NU-12 min sample is shown in Fig. 2c. This image clearly shows that the Au nanospikes growth can be considered as an even process over the whole QCM sensitive layer. The side view image of Au-NU-12 min is shown in Fig. 2d which provides further insight into the growth and formation of the packed urchin morphologies. The nanospikes are observed to start their growth on the e-beam evaporated semispherical Au shell of the Au-MNM samples. Due to the better access to the electrodeposition solution and more intense electrical field on the top of the Au-MNM, the spikes starts growing from the top-most area of these semispherical shells. Furthermore, as the structures have highly ordered packing arrangement in the monolayer formed, the nanospikes tend to point outwards, near-perpendicular to the horizontal plane of the electrodes.

The X-ray diffraction (XRD) pattern is presented in the Supplementary information (Fig. S2) and shows the crystallographic nature of the Au-NUs (Au-NU-6 min in this case). The XRD patterns could be indexed based on the face-centered cubic (FCC) structure of the polycrystalline gold (JCPDS: 04-0784). The results showed similar XRD patterns for all developed substrates.

The cyclic voltammogram (CV) of the Au^3+^/Pb^2+^ electrolyte used to deposit the Au-nanospikes directly on the Au-MNM is shown Fig. 3a. The Pb^2+^ ions work as an inorganic shape-directing additive for the growth of nanospikes in order to form the NU structures while it does not alter the surface chemistry of the deposited gold^42^,^54^. The deposition of the Au nanospikes was carried out at a constant potential (0.05 V) for periods of 6, 8, 10, 12 and 15 minutes, the current profiles (current-time curve) of which are presented in Fig. 3b. It can be observed that the current is increased and reaches stable state following the first 30 seconds of deposition, where the current is observed to reach a constant value ranging from −250 to −50 μA, depending on the sample. This fast transition to the steady stage is attributed to the uniform growth of nanospikes forming the NU nanostructures, the size of which depends on the deposition period. The electrochemically active surface area of the Au-NUs and Au-control were determined by using the linear sweep voltammetry (LSV) technique that was developed by Rand and Woods^42^,^54^,^55^. This involved recording the CVs for each sample at 100 mVs^−1^ in a 1 M H_2_SO_4_ solution vs Ag/AgCl auxiliary electrode. The CVs for the Au-control and Au-NU-6 min samples are shown in Fig. 3c. The increase observed at the cathodic peak (ca. 0.93 V) is indicative of oxide removal that was formed at the electrode surface during the forward sweep. The onset for this oxide formation is found to be at ~0.98 V and ~1.2 V for the Au-NU-6 min and Au-control, respectively. Similarly the CVs for the Au-MNM, Au-NU-8 min, Au-NU-10 min, Au-NU-12 min and Au-NU-15 min presented in the Supplementary information (Fig. S3) also show lower onset potentials for oxide formation thereby indicating that highly active Au nanostructures are present on the modified Au-NU surfaces. The shoulder observed at ca. 1.1 V before the main oxidation peak further confirms that the Au-NU-6 min have active surface defect sites^54^. When the active electrochemical surface areas of the samples were calculated using the reduction peak, it was observed that the Au-NU-6 min sample had over 6 times the surface area of Au-control sample. The difference in surface area is observed to increase with increasing electrodeposition time (see Supplementary information, Fig. S3) with the 15 minute electrodeposition of nanospikes (Au-NU-15 min) having ~10 times the surface area of the Au-control sample and more than 25 times higher than the geometrical surface area as shown in Fig. 3d. High surface area based gold nanostructures are of particular interest in numerous applications such as catalysis, batteries and bio-sensors^56^.

The fact that the developed Au-NUs are easily synthesized as highly ordered nano-arrays directly on a thin-film and can cover large active surface areas; they could be excellent candidates for sensing applications. Among the numerous possible applications, the developed Au-NUs were used to address a globally urgent and important issue (*i.e.* detection of toxic mercury vapor) in order to demonstrate their chemical sensing performance. Figure 4 shows the Au-NU based sensors’ response toward Hg^0^ vapor at 75 °C as well as its selectivity performance when tested toward Hg^0^ vapor with/without the presence of common interferent gases (*i.e.* H_2_O, NH_3_, Ethyl-M, MEK, Ac-ald and DMDS, as defined in the supporting information) found in industrial effluents. The sensitivity of the Au-NU based sensors toward Hg^0^ vapor is found to increase with increasing nanospike size, up to an electrodeposition time of 10 mins (Fig. 3a,b). It is observed that any electrodeposition beyond 10 minutes did not significantly increase the sensor sensitivity. A comparison between Au-control and Au-NU-12 min reveals that the modified sensor has more than 5 times the sensitivity of the Au-control QCM. This sensitivity increase is more apparent (15.75 times the sensitivity) when the sensors are operated at 30 °C (see Supplementary information, Fig. S4) even though Au-NU-12 min has only ~8.5 times the surface area of the Au-control based Hg^0^ vapor sensor. The presence of crystal lattice as well as surface topology defects are well known to be sorption sites for Hg^0^ vapor^20^,^57^,^58^. The relatively higher response magnitude of the Au-NUs toward Hg^0^ vapor is postulated to be due to the formation of such defect sites during the electrodeposition process when forming the nanospikes^42^. Therefore, although the Au-NUs was expected to have an even higher affinity toward Hg^0^ vapor due to increased number of defect sites, as noted in the Supplementary information, the Au-NU-15 min based QCM was dampened due to extensive growth of the nanospikes on the Au-MNM and so could not be tested. Regarding the sensing and adsorption-desorption of Hg^0^ on the gold surface, when fresh QCMs are exposed to Hg^0^ vapor, the first Hg^0^ pulse needs to be used as a pretreatment procedure (This is shown in Fig. S5). This process forms the initial Au-Hg amalgam layer on the surface of the Au film on QCM device. A portion of the initial frequency drop observed in the 1 hour Hg^0^ exposure step for both Au-control and Au-NU-12 mins is related to the formation of saturated amalgam layer on the surface of the Au structures. This portion is not recovered during the 1 hour N_2_ flushing and generation period since the operating temperature is maintained constant to enable chemical sensing experiments. The formation of this amalgam layer helps the surface to be saturated therefore allowing mercury atoms to only loosely adsorb to the surface during the Hg^0^ exposure event. As the regeneration process is introduced, these surface adsorbed Hg atoms do not have enough time to diffuse into the Au bulk and so most of the adsorbed Hg atoms desorb from the surface, thereby regenerating the surface. However, some diffusion of Hg into the Au surface is not avoidable due to Hg concentration gradient between the surface and bulk of the Au QCM electrode and so 100% regeneration can be a challenge. At higher temperatures, the Hg^0^ vapour pressure is higher and thus the desorption process occurs more readily. This is one of the main reasons for achieving better recovery at higher operating temperatures. Another important feature which is obtained by the formation of amalgam layer is the high reproducibility that can be achieved as shown in the Supporting information, Fig. S5. The Sauerbrey equation, which is a linear relationship between the frequency change of the QCM with that of the mass deposition, was used to determine the mass of Hg^0^ that was adsorbed and desorbed from the QCM surface during each pulse^59^ and presented in the right axis of Fig. S5. It can be observed that the sensor exhibited similar response magnitudes for each repeated concentration of Hg^0^ vapor tested and thus it operates more as an on-line sensor. In order to confirm that the developed Au-NUs based sensor response is solely due to their selective interaction with Hg^0^ vapor, they were tested toward six different interferent gases (listed in the Supplementary information, Table S1) with and without the presence of Hg^0^ vapor concentration of 3.26 ± 0.05 mg/m^3^ (Fig. 4c). The type and concentration of the interferent gases were specifically chosen to include those known for their high affinity towards Au surfaces as well as those commonly found in industrial effluents^42^. In order to show the extent of each sensor’s accuracy, solid magenta lines representing Hg^0^ vapor concentration of 3.26 mg/m^3^ is drawn in each panel. It can be observed that the sensor performance of the Au-NUs increased with increasing electro-deposition time and subsequent nanospikes size. The best accuracy was observed from the Au-NU-12 min based Hg^0^ vapor sensor (see the Supplementary information, Table S2) which showed 89% and 98% accuracy at ±10% (±0.33 mg/m^3^) and ±15% (±0.49 mg/m^3^) tolerance values, respectively. This excellent accuracy was further complemented with ~96% precision. The response time (based on 90% of full response magnitude) was estimated to be similar for all developed Au-NUs based sensors at approximately 48 minutes. Remarkably, the calculated LoD of the developed Au-NUs were found to be several fold lower than the Au-control based Hg^0^ vapor sensor with the largest tested Au-NU having the lowest LoD. That is, the Au-control and Au-NU-12 min based QCMs were found to have LoD of 149 and 32 μg/m^3^ at 75 °C, respectively. Based on the sensing data collected at 30 °C, the Au-NU-12 min showed and LoD of 16 μg/m^3^ which is the lowest LoD reported on QCM based Hg^0^ sensors reported as listed in Table 1. The relatively higher response magnitude and low LoD obtained with the developed Au-NUs as compared to other structures reported in literature is thought to have arisen from the presence of relatively large number defect sites (having high affinity toward Hg^0^ atoms) homogeneously formed throughout the whole sensitive layer due to growth of seamless spikes on ordered closely packed monolayer of Au-MNM. Furthermore, it was found that the Au-NUs based sensors had more than 90% recovery following each sensing event, without any external energy input thereby demonstrating their reusability over long periods of time. The results indicate that the developed Au-NUs based QCM sensors have high sensitivity and repeatability as well as being highly accurate toward determining low concentrations of Hg^0^ vapor in the presence of various interferent gas species. In addition to good sensitivity and selectivity, it is noteworthy that previous studies^60^,^61^ have shown Au-QCM based Hg sensors can have lifetimes exceeding 1 year period before their signal-to-noise ratio is reduced to levels where the sensor is deemed unusable. This has been shown through long-term experimental analyses and theoretical calculations based on the stable amalgam that can be formed at the different operating temperatures. These results suggest that Au-NUs can be used as a potential future generation mercury sensor technology for numerous industrial applications.

To summarize, for the first time we have developed a facile method for producing ordered monolayer nano-urchin gold nanostructures (Au-NU) with highly tunable sizes, thereby greatly expanding the possibility of devices that can be developed for various applications such as photonics, bio-chemical sensing and catalysis. The Au-NU were shown to encompass enhanced electrochemical activity over the control Au substrate. In order to demonstrate one to the Au-NU vast potential applications, the developed Au-NUs were formed on the electrodes of transducers (QCM) to selectively detect low concentrations of mercury vapor. It was found that by increasing the size of the nanospikes on the Au-NUs, the sensitivity and selectivity of the sensor device can be enhanced. The results showed that the Au-NU-12 min based QCM had the best performance for QCM based Hg^0^ vapor detection, displaying the highest sensitivity reported to date, 98% accuracy, 92% recovery, 96% precision (repeatability) and a limit of detection of of 32 μg/m^3^ toward Hg^0^ vapor at 75 °C. The results demonstrate the excellent activity of the developed materials which can be applied to a range of applications due to their long range order, tunable size and formation of Au nano-urchin structures directly on thin-film substrates.

## Methods

### Chemicals

The chemicals were purchased from Sigma Aldrich. All chemicals were used as received. The quartz substrates (AT-cut, optically polished, 10 MHz resonant frequency, Ø = 7.5 mm) were purchased from AATA in Japan.

### Polystyrene Nanoparticle Synthesis

The dispersion polymerization method used in this study to synthesize polystyrene nanospheres (PSNSs) is described elsewhere^20^,^62^. Briefly, a three neck round bottom flask was set-up under N_2_ atmosphere, containing 18 mL ethanol/water solution (20% water content) containing 800 mg of Polyvinylpyrrolidone (PVP) and a 2 ml volume of styrene was injected into the flask. The solution was heated and stirred at 70 °C and 1500 rpm, respectively. Another solution with 28 mg of initiator (Azobisisobutyronitrile (AIBN)) dissolved in ethanol (20 mL) was also prepared separately. This solution was then added to the three neck bottle thereby starting the polymerization reaction which was carried out for a period of 24 hours. The produced PSNS were mono-dispersed and had a size distribution of 500 ± 50 nm. The formed colloids were washed several times and finally dispersed in 40 mL ethanol.

### Transducer Fabrication

The quartz crystal microbalance (QCM) transducers were fabricated through e-beam deposition of a 300 nm Ti layer on each of the two faces of quartz substrates in order to form the Ti electrodes (Ø = 4.5 mm). Employing Ti electrodes was mainly due to the metal’s good adhesion properties to quartz as well as having no affinity toward (Hg^0^) vapor^63^.

### Colloidal Monolayer Formation

The polystyrene monodispersed nanosphere monolayer (PS-MNM) based Au (Au-MNM) was fabricated using the procedure described elsewhere^20^. Briefly, a monolayer of PSNS was formed by injecting a ~10 μL of the PSNS solution on the water/air interface formed in a glass Petri dish. A ~2 drop (20 mg/mL) SDS (sodium dodecyl sulfate) was then added to the interface to form close-packed monolayers of the PSNS colloids. This monolayer was transferred directly on the Ti electrodes of the QCM surface. The modified QCMs were dried with N_2_ gas prior to depositing (Balzers, BAK 600) a 100 nm layer of Au thereby forming Au-MNM based QCM transducers.

### Au based nano-urchin (Au-NU) formation

The Au-MNM were converted to Au-NUs of various sizes following a one-step electrodeposition (CH Instruments, CHI 760C) of gold nanospikes for various periods of time, directly on the electrodes of the QCM transducers. The different sizes of Au-NUs were formed by electrochemical depositing of Au-nanospikes for a period of 6, 8, 10, 12 and 15 minutes. The electrolyte solution used contained 2.718 g/L hydrogen tetrachloroaurate(III) tri-hydrate and 0.177 g/L lead(II) acetate tri-hydrate. The working electrode was Au-MNM while Ag/AgCl (3M KCl) and graphite served as auxiliary and counter electrodes, respectively. A constant voltage deposition of 0.05 V was used and was based on the electrolyte CV. Further specifics regarding nanospike deposition can be found elsewhere^42^,^54^.

### Surface Characterization

The modified substrates were first characterized by scanning electron microscopy (SEM) using a Nano-SEM instrument. XRD measurements were performed using Bruker D8 Discover micro diffraction system operating at 40 kV and 40 mA with Cu Kα radiation. The instrument came with a general area detector diffraction system (GADDS). Cyclic voltammetry (CV) of Au-control and Au-NUs were performed in 1 M H_2_SO_4_ at 100 mVs^−1^ in order to determine the active electrochemical surface areas (ESA).

### Hg^0^ Sensing Experiments

The experimental procedures detailing transducer characterization and Hg^0^ vapor sensing are included in the Supplementary Information Section.

## Additional Information

**How to cite this article**: Sabri, Y. M. *et al*. Ordered Monolayer Gold Nano-urchin Structures and Their Size Induced Control for High Gas Sensing Performance. *Sci. Rep.* **6**, 24625; doi: 10.1038/srep24625 (2016).

## Supplementary Material

Supplementary Information

## Figures and Tables

**Table 1:** A comparison between Au-Nu with other reported QCM sensors for Hg^0^ vapor detection at 35 °C.

**Sensing material**	**LOD (μg/m**^**3**^)	**Sensing Temp. (**°C)
Au film^64^	537	NR
Au film^65^	80	NR
palladium (II) chloride and tetra-hydroxyethyl-ethylene-diamine (THEED)^66^	1790	NR
Au coated nano templates of molybdenum oxide (MoO_3_)^67^	4390	36
Ag film^58^	1020	40
Polypyrrole (PPy) nanofibers loaded with PPy/Pd(O_2_CCH_3_)_2_)/(PPy/Pd(NO_3_)_2_)^68^	1020	30
Au nanoprisms^61^	21	30
Ni/Au galvanically replaced thin films^63^	103	90
Pd/Au galvanically replaced thin films^69^	34.2	28
Au nanospikes^42^	22	28
Au nanosphere monolayers^20^	27	30
Au/Ag galvanically replaced nanowire monolayers^70^	39	30
	53	75
*Au-Nu monolayer (This work)*	16	30
	32	75

NR: Not reported.
