# Supplementary material for: Ordered Monolayer Gold Nano-urchin Structures and Their Size Induced Control for High Gas Sensing Performance
Source: Sci Rep. 2016 Apr 19;6:24625. doi: 10.1038/srep24625 (PMC4835752; doi:10.1038/srep24625)
Supplement: Supplementary Information [file srep24625-s1.pdf]

Electronic Supplementary Information (ESI)

Ordered Monolayer Gold Nano-urchin Structures and Their Size Induced Control for High Gas Sensing Performance

Ylias M. Sabri, Ahmad Esmaielzadeh Kandjani, Samuel J. Ippolito and Suresh K. Bhargava**

Centre for Advanced Materials and Industrial Chemistry (CAMIC), School of Applied Sciences, RMIT University, GPO Box 2476V, Melbourne, VIC 3001 (Australia).

Email: ahmad.kandjani@rmit.edu.au; suresh.bhargava@rmit.edu.au ; Phone: +61 3 99252330

SUBJECT AREAS: Physical Sciences - Materials Science; Nanoscience and Technology

Experimental details

Transducer Characterization: The QCM quality factors (or Q-factor) were determined using an Agilent E5100A network analyzer in order to assess their suitability for being employed as a gas sensor. The Q-factor is an indication of the sensor performance as it represents the stored to dissipated energy ratio. A minimum Q-factor of 2500 was required for the QCM transducers to be operated in the designed sensor chamber. The Au-NU-15min failed to meet the Q-factor requirements for the system and so was not employed as a Hg^0 vapor sensor.

Hg^0 Vapor Sensing: The gold nano-urchin (Au-NU) based QCMs and the unmodified Au-film (Au-control) based QCMs were tested toward various concentrations (0.21, 0.31, 0.45, 0.64, 0.93, 1.27, 1.74, 2.38, and 3.26 ± 0.05 mg/m^3) of elemental mercury (Hg^0) vapor at 30 and 75 ± 1°C. The 30°C operating temperature condition was chosen as it was the lowest stable temperature that could be controlled. However, 75°C was preferred and maintained as it was most applicable in industrial processes[1] and the relatively higher temperature can enhance the selectivity and recovery performance of the QCM based sensor toward Hg^0 vapor.[2] Hg^0 permeation tubes purchased from VICI, USA were used to generate highly controlled concentrations of Hg^0 vapor of different magnitudes. The magnitudes of the concentrations of Hg^0 vapor generated were validated using an acidic potassium permanganate ($KMnO_4$) impinger train (similar to those used in US-EPA's OH method) in conjunction with inductively coupled plasma mass spectroscopy (ICP-MS, ShieldTorch System, HP4500 series 300). The center frequency changes of the QCMs were monitored throughout the tests using research quartz crystal microbalance (RQCM, Maxtek) units which have frequency resolution of ±0.03 Hz for each of the three measuring ports.

The procedure used for all sensing measurements involved the exposure of the sensors to a gas stream containing a mix of dry N_2 with/without the presence of interferent gases and/or Hg^0 vapor for 1 hour followed by a regeneration step where only dry N_2 was exposed for another 1 hour period. This

whole 2 hour procedure is referred to as a pulse. The total gas flow rate was kept constant at 200 sccm throughout the experiments. The flow rates of each gas were controlled using mass flow controllers purchased from MKS instruments in USA. The interferent gas types and concentrations tested are listed in **Table S1** and included numerous volatile organic compounds (VOCs) commonly present in most industrial processes[3] and gas species that can potentially have cross-sensitivity issues when employing Au sensitive layers.[4-14]

Table S1. Interferent gases and levels exposed to Au-NU based QCMs during the selectivity tests.

**Interferent gas**	**Concentration**
Humidity (H_2O)	27.2 g/m^3
Ammonia (NH_3)	384 ppm
Acetaldehyde (Ac-ald)	304 ppm
dimethyl disulfide (DMDS)	5.01 ppm
Ethyl mercaptan (Ethy-M)	2.61 ppm
methyl ethyl ketone (MEK)	40.1 ppm

The humidity level of 27.2 g/m^3 was generated using a relative humidity generator purchased from V-Gen, InstruQuest. This humidity level used was calculated to be equal to testing a stream of 100 %*RH* at 50 °C, once the 1:3 dilution factor employed is accounted for.

The accuracies of the fabricated sensors were estimated using the fitted calibration curves (Equation 1). The calibration curve that fit best for the developed sensors is known as the three parameter Langmuir-Freundlich model[15] or the loading ratio correlation (LRC).[16] The parameters Δf_m, β and η in the equation represent the equation constants and the fit in the data produced $R^2 > 0.99$ for all the fitted curves at 75°C. The LRC fits were used to convert the reported sensor response toward Hg^0 vapor during the selectivity tests, into Hg^0 vapor concentrations as reported in **Figure 3c**.

$$\Delta f = \frac{\Delta f_m \beta C^\eta}{1 + \beta C^\eta} \qquad (1)$$

References

1 Levlin, M., Niemi, H. E. M., Hautojärvi, P., Ikävalko, E. & Laitinen, T. Mercury adsorption on gold surfaces employed in the sampling and determination of vaporous mercury: a scanning tunneling microscopy study. *Fresenius J. Anal. Chem.* **355**, 2-9 (1996).

2 Sabri, Y. M., Kandjani, A. E., Ippolito, S. J. & Bhargava, S. K. Nanosphere Monolayer on a Transducer for Enhanced Detection of Gaseous Heavy Metal. *ACS Appl. Mater. Interfaces* **7**, 1491-1499 (2015).

3 Mullett, M., Tardio, J., Bhargava, S. & Dobbs, C. Removal of mercury from an alumina refinery aqueous stream. *J. Hazard. Mater.* **144**, 274-282 (2007).

4 Bilic, A., Reimers, J. R., Hush, N. S. & Hafner, J. Adsorption of ammonia on the gold (111) surface. *J. Chem. Phys.* **116**, 8981-8987 (2002).

5 Tanida, K. & Hoshino, M. Contiuous determination of mercury in air by gold amalgamation and flameless. *The Rigaku Journal* **7**, 35-40 (1990).

6 Finklea, H. O., Avery, S., Lynch, M. & Furtsch, T. Blocking oriented monolayers of alkyl mercaptans on gold electrodes. *Langmuir* **3**, 409-413 (2002).

7 Rocha, T. A. P., Gomes, M. T. S. R., Duarte, A. C. & Oliveira, J. A. B. P. Quartz crystal microbalance with gold electrodes as a sensor for monitoring gas-phase adsorption/desorption of short chain alkylthiol and alkyl surfides. *Anal. Commun.* **35**, 415-416 (1998).

8 Richton, R. E. & Farrow, L. A. Adsorption kinetics of ammonia on an inhomogeneous gold surface. *J. Phys. Chem.* **85**, 3577-3581 (2002).

9 Kay, B. D., Lykke, K. R., Creighton, J. R. & Ward, S. J. The influence of adsorbate--absorbate hydrogen bonding in molecular chemisorption: NH_3, HF, and H_2O on Au(111). *J. Chem. Phys.* **91**, 5120-5121 (1989).

10 Surplice, N. A. & Brearley, W. The adsorption of carbon monoxide, ammonia, and wet air on gold. *Surf. Sci.* **52**, 62-74 (1975).

11 Nuss, H. & Jansen, M. [Rb([18]crown-6)$(NH_3)_3$]Au$\cdot NH_3$: Gold as Acceptor in N-H$\cdots Au^-$ Hydrogen Bonds. *Angew. Chem., Int. Ed.* **45**, 4369-4371 (2006).

12 de Vooys, A. C. A., Mrozek, M. F., Koper, M. T. M., van Santen, R. A., van Veen, J. A. R. & Weaver, M. J. The nature of chemisorbates formed from ammonia on gold and palladium electrodes as discerned from surface-enhanced Raman spectroscopy. *Electrochem. Commun.* **3**, 293-298 (2001).

13 Mirsky, V. M., Vasjari, M., Novotny, I., Rehacek, V., Tvarozek, V. & Wolfbeis, O. S. Self-assembled monolayers as selective filters for chemical sensors. *Nanotechnology* **13**, 175-178 (2002).

14 Meyer, R., Lemire, C., Shaikhutdinov, S. K. & Freund, H. Surface chemistry of catalysis by gold. *Gold Bull.* **37**, 72-124 (2004).

15 Yao, C. Extended and improved Langmuir equation for correlating adsorption equilibrium data. *Sep. Purif. Technol.* **19**, 237-242 (2000).

16 Kapoor, A., Ritter, J. A. & Yang, R. T. An Extended Langmuir Model for Adsorption of gas Mixtures on Heterogeneous Surfaces. *Langmuir* **6**, 660-664 (1990).

Supporting Figures

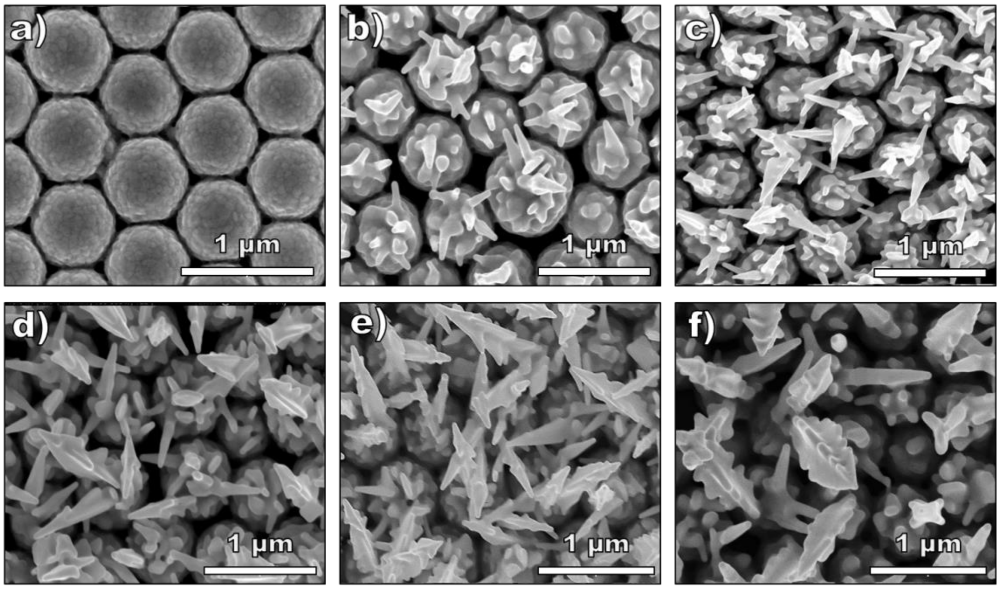

Figure S1. SEM images representing **a)** close-packed Au-MNM, **b)** Au-NU-6min, **c)** Au-NU-8min, **d)** Au-NU-10min, **e)** Au-NU-12min and **f)** Au-NU-15min.

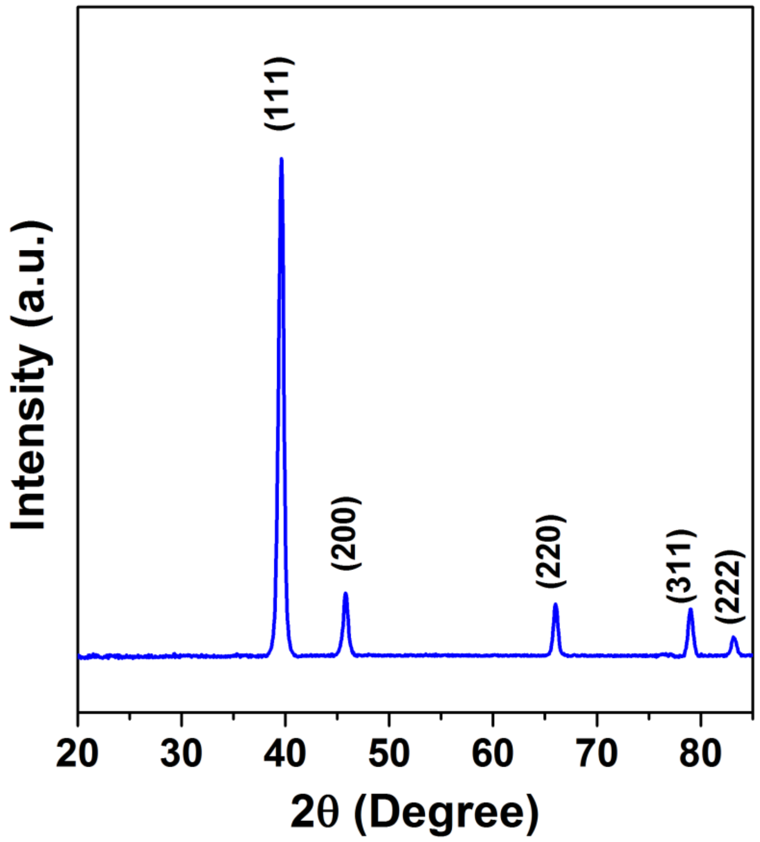

Figure S2. XRD patterns of Au-NU-6min.

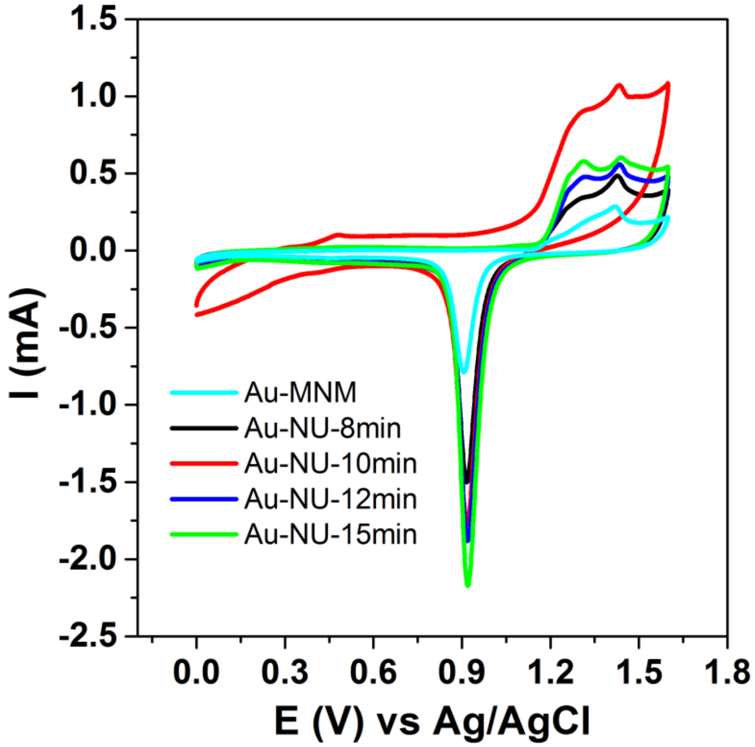

Figure S3. Electrochemical characterization data showing the linear sweep voltammograms (LSVs) obtained at 100 mV s^{-1} in 1 M H_2SO_4 solution for the Au-MNM, Au-NU-8min, Au-NU-10min, Au-NU-12min, and Au-NU-15min. The reduction peaks of the CVs (recorded in 1 M H_2SO_4) were used to estimate the active electrochemical surface area (ESA) for each sample. The geometric surface area of each substrate was 0.196 cm^2.

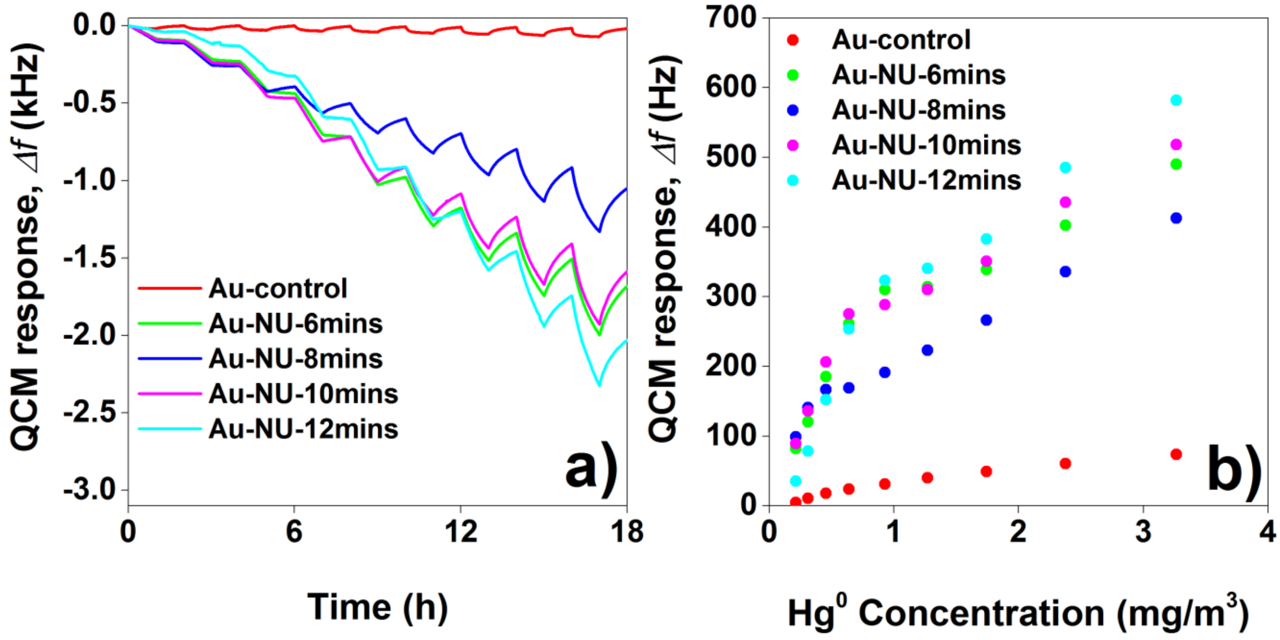

Figure S4. Au-control and modified (Au-NUs) based QCMs' **a)** dynamic response and **b)** response magnitudes toward Hg^0 vapor at 30°C. The dynamic response was obtained when the QCMs were exposed toward Hg^0 vapor concentrations in sequential order from lowest to highest concentrations (i.e., 0.21, 0.31, 0.45, 0.64, 0.93, 1.27, 1.74, 2.38, and 3.26 ± 0.05 mg/m^3).

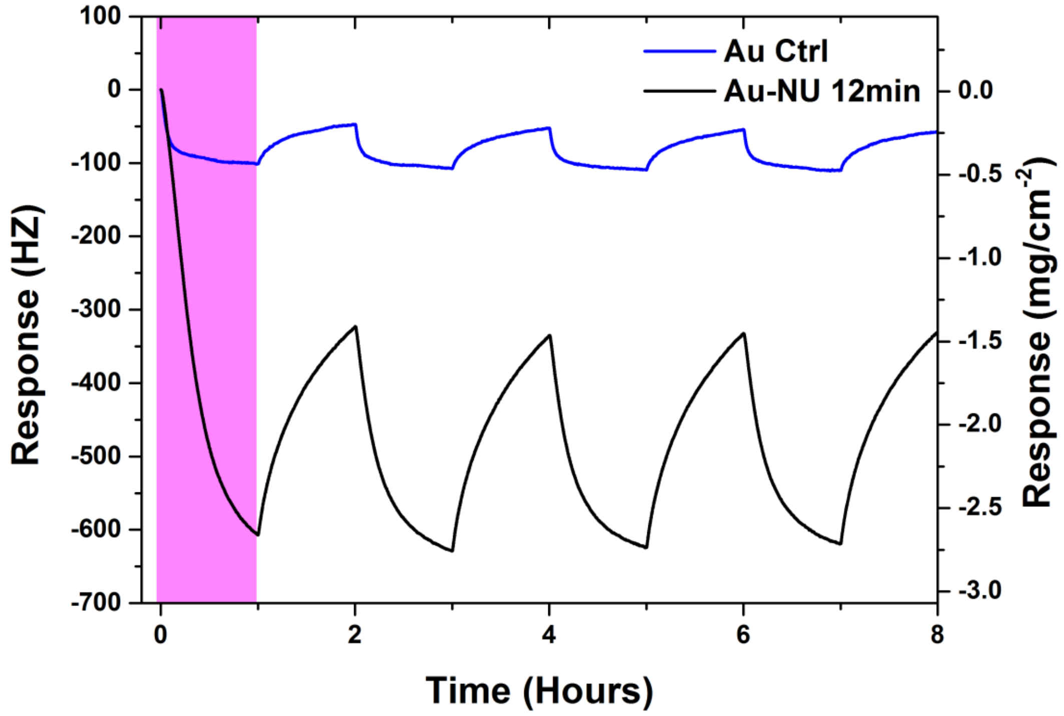

Figure S5. Dynamic response of untreated samples toward Hg^0 vapor concentration of 3.26 ± 0.05 mg/m^3 for Au-control and Au-NU-12min sensors over 4 pulses. The shaded magenta area shows the pretreatment step which renders the QCM to be highly reproducible when employed for Hg^0 vapor sensing applications.

Table S2 Au-control and modified sensor performances such as response time (t_{90}), detection limit (LoD), extent of recovery, repeatability and accuracy at 75°C. The data presented is from the sensors' response when they were exposed to 3.26 mg/m^3 of Hg^0 vapor. The accuracy is reported for a ±15% tolerance which is better than the ±20% tolerance allowed by the EPA in order to validate any new sensor against the OH method. The sensors' repeatability was calculated from their response magnitudes toward 10 consecutive pulses of Hg^0 vapor (3.26 mg/m^3).

	Au-ctrl	Au-MNM	Au-NU-6min	Au-NU-8min	Au-NU-10min	Au-NU-12min
t_{90} (min)	24	29	48	47	48	47
LoD (µg/m^3)	149	130	35	37	32	32
Recovery (%)	91	92	92	92	90	92
Accuracy (%)	45	97	33	44	89	98
Repeatability (%)	75	84	85	90	95	96
